# Interactive Family Dynamics and Non-suicidal Self-Injury in Psychiatric Adolescent Patients: A Single Case Study

**DOI:** 10.3389/fpsyg.2017.00046

**Published:** 2017-02-06

**Authors:** Michela Gatta, Marina Miscioscia, Marta Sisti, Ilaria Comis, Pier Antonio Battistella

**Affiliations:** ^1^Childhood Adolescence Family Unit, ULSS 16 – University of PaduaPadua, Italy; ^2^Department of Women’s and Children’s Health, University of PaduaPadua, Italy; ^3^Department of Developmental and Social Psychology, University of PaduaPadua, Italy

**Keywords:** Lausanne Trilogue Play, non-suicidal self-injury, family interactions, adolescence, family functioning

## Abstract

Non-Suicidal Self-Injury (NSSI) is a common, multifaceted phenomenon among adolescents. Recent researchers have shown that a number of psychological and psychiatric correlates are implicated in the onset/repetition of NSSI, but those previous studies did not directly observe the family interaction patterns of this clinical population. In this paper, the quality of family interactions was observed using the Lausanne Trilogue Play procedure to deepen the specific interactive dimensions associated with NSSI in adolescents. The results of a single case study showed a lack of positive emotional exchanges, a parenting style expressing hostility, a high level of control and difficulties in triangulation. Through this method, the authors show that a better understanding of the role of family interactions is crucial and could improve the assessment and treatment of Non-Suicidal Self-Injurious behaviors. Research and clinical implications are discussed.

## Introduction

Non-Suicidal Self-Injury (NSSI) is a common and increasing phenomenon among teenagers. Clinical literature shows different types of both individual and group treatments for self-harming adolescents (Gatta et al., 2010, 2014; Mehlum et al., 2016; Steuwe et al., 2016), but none of the previous research include the study of family interventions. Indeed, nowadays only a few published studies have focused on family relationships as part of adolescent NSSI researchers, and several weaknesses have also been identified in the limited literature available, including the use of self-report measures regarding the quality of the parent-child relationship. The affective bounds between the parents and the child/adolescent are usually investigated (Laukkanen et al., 2009; Hankin and Abela, 2011; Mannarini and Boffo, 2014). Strong associations have been identified between NSSI and insecure or traumatic attachment relationships, separations and bereavements in infancy, dysfunctional parent-child relationships, and excessively critical parenting behavior (Heath et al., 2008; Laukkanen et al., 2009; Plener et al., 2009). Di Pierro et al. (2012) highlight that an inadequate relationship with the mother is linked to both the existence and the seriousness of the NSSI act, while the relationship with the father affects exclusively the behavior intensity. In other words, adolescents are more likely to frequently engage in NSSI acts when the relationship with their father is characterized by poor communication, rare acts of love on the father’s end and admiration from the adolescent toward his/her father.

When focusing on family dynamics during the adolescence period, Lineahn (1993) demonstrated that the families of self-harming adolescents are characterized by dysfunctional parenting in a setting where children’s personal experiences and emotions communications are all too often ignored, trivialized or punished, instead of being encouraged and supported. Subsequent studies will confirm the link between emotional dysregulation, an invalidating family environment and the NSSI onset and persistence (Crowell et al., 2008; Kaess et al., 2012; Gatta et al., 2016).

In addition to the nature of the family structure and the quality of parent–child relations, a fundamental variable considered in the NSSI literature concerns the parents’ behavior. Tschan et al. (2015) confirmed that the onset and persistence of NSSI could be considerably influenced by an absence of warmth and support, as well as by a marked hostility and a severely critical parenting approach. The authors also found that NSSI adolescents’ parents reported higher levels of parental stress and lower levels of parental satisfaction than other parents. The NSSI adolescents’ mothers also revealed more symptoms of depression, anxiety and stress than the mothers whose adolescents do not suffer from NSSI. Hilt et al. (2008) noted that self-harming adolescents’ relationships with their fathers were characterized by poor communications and rare, sporadic demonstrations of affection, alongside strong sentiments of admiration and adoration on the adolescents’ part – a situation that paved the way to severe mechanisms of idealization which can cause a discrepancy between the real parent–child relationship and an imaginary parent–child relationship.

Although the existing literature shows interest regarding NSSI and the relationship between NSSI and family dynamics, the methodology used to assess this issue remains limited. As a matter of fact, only a few studies have attempted a systematic observation of family interactions in this particular population so far, assessed via self-report measures. Literature on family interactive patterns and its influence on the child development has extensively increased, which helps providing new observational tools with a consistent coding system. Elisabeth Fivaz-Depeursinge and Corboz-Warnery (1999) have created an innovative semi-standardized play situation called the Lausanne Trilogue Play (LTP): the mother, father and child complete a non-distressing task in order to assess the interactive family behaviors. This theoretical approach has been used in several studies to stress the impact of family interactions quality on both the cognitive and the emotional child development (e.g., Favez et al., 2012). This approach has been recently applied to clinical populations proving how this instrument can identify the dysfunctional patterns of interactions and consequently suggesting its use as a diagnostic and intervention tool.

The present paper provides a clinical exemplification by means of a case report on the application of the LTP (Fivaz-Depeursinge and Corboz-Warnery, 1999) in order to observe family interactions in a family with an adolescent patient engaging in NSSI.

## Maria’S Family

We met Maria (17 years old) and her parents at the Neuropsychiatric Unit for Children and Adolescents (*Struttura Complessa Infanzia Adolescenza Famiglia* SCIAF-ULSS 16) in Padua (Italy), where they had been referred for a diagnostic assessment because Maria’s behavior had worried her schoolteachers and her pediatrician. The diagnostic workup suggested an Affective Syndrome (ICD-10, F39 World Health Organization [WHO], 1992) with NSSI. The girl’s family history featured frequent separations between Maria and her father, out of economic necessity, and previous depressive problems in the mother. Maria had shown signs of separation anxiety in childhood, particularly when she started school, and difficulties in establishing relationships with peers. Her self-harming behavior had appeared after her brother was born, when Maria was 13 years old. At that time, she would harm herself once or twice per month, as reported by Maria during the diagnostic process, and the wounds were made with a razor blade on her arms and shoulders. Alongside these issues, Maria presented extreme sadness and hopelessness, episodes of irritability and weeping. She also reported a general loss of interest, a low self-esteem and difficulties interacting with peers. She didn’t show any changes in her eating or sleeping habits, they both remained satisfying. As reported by Maria, over the past few months, her school performance, despite being sufficient, worsened as she has difficulty concentrating. During the diagnostic assessment, the parental satisfaction evaluation showed a low level in the mother and an average level in the father. This evaluation was made with the Family Empowerment Scale (FES-Koren et al., 1992) that measures the self-perception of parental skills.

## Methods And Procedure

This clinical case was part of a larger longitudinal study conducted at the Neuropsychiatric Unit for Children and Adolescents run by the Italian national health services in Padua (Italy), where the LTP procedure is used not only as a diagnostic tool, but also as part of the treatment assisted by video feedback (VF). Our aim is to explain how the LTP procedure can help clinicians to detect dysfunctional family patterns, a key aspect of the NSSI’s families. For the purposes of the treatment provided, parents work with a psychotherapist in fortnightly sessions, with a VF application. This procedure is designed to elucidate the micro frame of selected dysfunctional family interactions and to promote functional changes. This approach is based on the assumption that a good working alliance with the two parents and the child is something to aim for, without considering it as an *a priori* motivation for the clinical intervention but as the primary goal (Gatta et al., 2009).

The setting has been specifically devised for adolescents (Ballabio et al., 2009) and consists of the family making plans for the adolescent’s birthday party, based on a four-part scenario relating to the four possible relational configurations in a triad: (1) 2 + 1, one parent is active with the adolescent and the other parent is only an observer; (2) 2 + 1, the two parents swap roles; (3) 3, all three actors discuss together; (4) 2 + 1, the parents discuss together and the adolescent becomes the observer. The procedure is recorded with a video camera and behavioral criteria are coded according to the FAAS manual^1^ (Family Alliance Assessment Scale 6.3; Lavanchy Scaiola et al., unpublished). The analysis of the clinical case reported below was conducted with all the LTP^2^ variables (e.g., the criteria for the variable Posture and Gaze aims to observe if the partners’ bodies are engaged or not in the interaction area –triangle- and then if everybody is oriented in a manner facilitating the interaction; e.g., for the Co-construction variable, coders evaluate if members co-construct the joint activity keeping the discussion together; if maladjustments are displayed in the role of the active parent and the sharing affect is measured through a micro analytic observation).

## Exclusion and Trivialized Affective Experience

The following descriptions are based on the behavioral coded criteria according to the FAAS manual.

At the beginning of the procedure, the parents took a moment to decide who should go first: the father decided how they should take turns, showing a commanding and controlling style. Despite the father’s managing style, the family remained on hold during this prelude, immediately revealing the two parents’ difficulty in sustaining each other and coordinating their actions. It was Maria who began the LTP task, acting as a regulating element that enabled the two parents to overcome the impasse of the prelude and start the task. Maria turned her face and upper body toward her mother, physically excluding her father. The mother remained seated, leaned against the back of her chair, leading her daughter to emphasize the relational configuration. Maria and her mother started to plan Maria’s birthday party in a cooperative atmosphere in which the girl adequately communicated her wishes and proposals, while her mother listened and co-constructed the activity. She happened to be emotionally detached and hardly ever genuine. The father listened very carefully and, on two occasions, caused a major disruptive interference that prompted Maria to say, “Shhh, be quiet,” to remind him that he was not to speak in this first configuration. This LTP segment shows the difficulty of Maria’s father to let his daughter and wife (the mother) discuss and decide together, while remaining in an observational role. As part of the arrangements for her birthday party, Maria asked her mother if the parents could stay away from the party. Her mother appeared to have difficulty in managing this request alone and, after a first attempt to change the subject, she agreed with little conviction, owed to Maria’s father body signals. Indeed, he showed clear signs of disapproval -movements of the head and low gaze- that the mother could see, unlike Maria, as the three participants were not facing each other. After agreeing on everything with her mother, it was Maria who moved on to the second part of the LTP by saying: “Now I can tell him.” In this second part of the task, Maria turned toward her father, who remained in the same position as before, with his body leaning forward. Maria’s body position included her mother in the interactive triangle (unlike the situation in the previous configuration). Her father started asking her to summarize what she and her mother had said, while keeping his gaze lowered and nodding. When Maria completed her brief account, her father exclaimed, “I don’t agree. I don’t want you to be left by yourself.” His words generated a reaction of disappointment and anger in Maria, though she remained composed and controlled. She tried to debate the point but her father was inflexible and, within seconds, the girl’s invalidated and trivialized emotions made her shut down (lowering her shoulders and gaze) and she started to weep, silently.

## Emotional Detachment and Lack of Cohesion

During the second part of the LTP, Maria’s mother remained in an appropriate position for her role as observer, but showed clear signs of emotional detachment in her motionless facial expressions. Before withdrawing, Maria tried to catch her mother’s eye, but her mother kept on fixing her look on the interaction space. In this part of the LTP, the cooperation between Maria and her mother, which excluded the father in the first part, was replaced with the cooperation between the father, in command, and the mother, who was emotionally detached. The activity shared by the father and daughter was no longer apparent, and there was no validation of the decision made before from either of the parents. At this point, the father turned to the mother and said: “What do you have to say Mummy?.” This marks the transition to the third part of the task: with her gaze lowered and a feeble tone of voice, the mother asked the father: “Why don’t you agree?.” The father, turning to Maria, gave some examples of possible catastrophic events, such as “a short circuit” or “a fire.” The mother was excluded, and the interaction developed again for a few seconds between the father and daughter. Maria started to cry, describing her feelings. This prompted the mother to make a timid effort to find a compromise, always speaking to her daughter and never to her husband, while wiping away a tear from her daughter’s cheek. This gesture did not seem to help the father understanding the girl’s state of mind. Instead, it created a covert conflict between the two parents, which was settled after a few seconds by Maria’s mother backtracking and adopting the father’s initial position.

There was a gradual deterioration in the quality of the activity, with Maria trying to explain to her parents why she was sad and what she needed. Albeit they moved on to a brief fourth part of the procedure, the father reiterated the reasons for his decision and the exchange between the two parents remained poor and lacking of authenticity. After a little while, Maria suggested to put an end to the task.

## Discussion and Therapeutic Implications

This case report gives an example of the LTP procedure efficiency to identify dysfunctional family patterns. In line with the previously mentioned literature, Maria’s family interactions are characterized by a lack of positive emotional communication and a parenting style expressing both hostility and a high level of control (Lineahn, 1993; Crowell et al., 2008). Maria seems to have an important role in the family’s relational setting, as a mediator, but also as a catalyst for her parental conflicts. The initial alliance between the mother and Maria turns out to be superficial and the mother happens to lack of awareness regarding Maria’s needs. The mother’s affective and relational abilities outline an emotional distance: by means of the LTP procedure, the clinicians could identify a low level of parental satisfaction, according to the measurements made during the diagnostic assessment through the FES, and the depression symptoms in the mother, which were subsequently assessed by other professionals and confirmed.

This picture is consistent with the study conducted by Hilt et al. (2008) and it underlines how an emotional distance expressed by the mother figure may create difficulties in the children capabilities to represent the internal affective states. As reported also by Di Pierro et al. (2012), the quality of the relationship with the mother proved to be the most important aspect linked to NSSI acts.

Concerning the relationship between Maria and her father, the observations on the father’s parental attitude revealed that it was based on an intrusive control and an overall difficulty in understanding and validating his daughter’s age-related needs. The father did not seem to develop a supportive role in the relationship with his daughter and he did not seem able to facilitate her emancipation. According to the literature, it appears to be specifically important to observe the quality of the father–daughter relationship as it influences the NSSI acts (Di Pierro et al., 2012) gravity. The LTP procedure helps to observe the relationship quality between the adolescent and each of his/her parent, but also the triangular communication.

With the identification of specifics interactive behaviors noticed during the LTP procedure, clinicians could show to parents some extracts and help them understanding what happened during those interactions in order to promote the changes. The LTP task, divided in four segments, allows the observation of each member’s resources and difficulties, and each interaction (dyadic versus triadic; marital couples versus co-parenting, etc.). Using this procedure as part of a diagnostic assessment enables such specific relational patterns to be identified and provides an opportunity to work on them in a therapeutic setting with the aid of the VF; it also explores the possibility to build a positive working alliance and give a caregiving experience to the parents (Gatta et al., 2011). Beyond the contents, the LTP procedure works as an illustration for the parents, to help them realize what kind of behaviors, or lack of affective containments, can produce discomfort in the adolescent, in a simple and playful situation such as the organization of the birthday.

Research on this topic needs to be extended, as for instance, case-control studies with different clinical populations presenting various psychiatric conditions, in order to understand if a specific interactive family pattern could be identified in families with NSSI adolescents. For both diagnostic and therapeutic purposes, it is fundamental to analyze the interactive relational dynamics involved, using a multi-method investigation that includes: the adolescent’s personality profile observation, the quality of the co-parenting alliance, the parental stress level, as well as the relations between attachment and alliance.

## Ethics Statement

Ethical Committee (CEP 204 SC) provided by ULSS 16 General Director in date 10/10/2013. Participants were given the research protocol explaining the project details: Titular of the treatment and relative finalities; Nature of data; treatment modalities; informations about participants rights. All these details follow a specific normatives that were detailed in the consensus protocol. The study involve minors, their parents sign the content procedure and LTP videotape in line with the privacy normative specific for this kind of study.

## Author Contributions

All authors listed, have made substantial, direct and intellectual contribution to the work, and approved it for publication.

## Conflict of Interest Statement

The authors declare that the research was conducted in the absence of any commercial or financial relationships that could be construed as a potential conflict of interest.

## Footnotes

1 The assessment of the interactive dimensions consist in 15 variables assessed with three point (from 1 “inappropriate” to 3 “appropriate”): Posture and gazes, Inclusion of partners, Role implication, Structure, Co-construction, Parental scaffolding, Family warmth, Validation, Authenticity, Interactive mistakes during activities, Interactive mistakes during transitions, Support, Conflicts, Involvement, and Self-regulation.
